# The effect of school science education on students’ climate literacy: a three-level meta-analysis

**DOI:** 10.3389/fpsyg.2026.1769772

**Published:** 2026-03-04

**Authors:** Junyuan Chen, Ying Ren, Yanru Yang

**Affiliations:** 1 Normal School of Vocational Techniques, Hubei University of Technology, Wuhan, China; 2 School of Education Science, Shanxi University, Taiyuan, China

**Keywords:** climate change, climate literacy, meta-analysis, science education, environmental psychology

## Abstract

**Background:**

Climate literacy is fundamental to addressing climate change. Science education has unique value in shaping students’ climate literacy. Given the divergence among single studies, it is essential to synthesize findings from multiple empirical investigations to comprehensively explore the impact of science education on students’ climate literacy, yet such research remains scarce.

**Objective:**

This study employed a three-level meta-analysis to examine the effectiveness of school science education in fostering students’ climate literacy.

**Methods:**

We systematically searched five databases for studies published up to May 2025, ultimately including 33 experimental and quasi-experimental studies (*N* = 8,044) from five continents. We conducted three-level meta-analysis of overall effects and moderation effects using the metafor package in R software.

**Results:**

The results indicated that school science education has a significantly positive and large effect on students’ climate literacy (*g* = 1.01, 95% CI [0.70, 1.33], *p* < 0.001, *k* = 60). Dimensional analyses showed significant positive effects on climate change cognition (*g* = 1.08, 95% CI [0.71, 1.45], *p* < 0.001, *k* = 46) and attitudes (*g* = 0.88, 95% CI [0.17, 1.60], *p* < 0.05, *k* = 11), with a larger effect for cognition. The effect on climate action was not statistically significant (*g* = 0.34, 95% CI [−0.63, 1.32], *p* > 0.05, *k* = 3). Discipline significantly moderated the effect of science education on climate literacy, whereas instructional strategy, educational level, and intervention duration did not.

**Conclusion:**

Our research indicates that school science education is an effective pathway for enhancing students’ climate literacy, though the impact varies across different science disciplines. However, given the considerable heterogeneity among the included studies and the limited number of studies and effect sizes in some groups, these findings should be interpreted with caution.

## 1 Introduction

Climate change induced by human activities has reached unprecedented levels, with its associated adverse ecological and societal effects intensifying annually. This trend renders the global task of addressing climate change increasingly urgent (WMO, 2025; IPCC, 2023). The latest decision of the United Nations Framework Convention on Climate Change (UNFCCC, 2023) in 2023 has reinforced the necessity for global coordinated responses and calls for systemic actions to mitigate climate risks across nations. Climate literacy serves as a cornerstone for mitigating the impacts of climate change (Kumar et al., 2023). Specifically, climate literacy refers to an understanding of how the climate system works, how human actions influence the climate, and how climate change affects humans and other components of the Earth system (USGCRP, 2024). Individuals who are climate-literate understand the fundamental principles of the Earth’s climate system and the available options for responding to anthropogenic climate change; they are able to critically evaluate information related to climate change and know where to find reliable sources of such information. In addition, they can respond to climate anxiety with constructive attitudes, communicate about climate change accurately and effectively, and make informed decisions related to climate issues. Climate-literate individuals are also able to integrate cognition, emotions, attitudes, values, and actions to address climate change (USGCRP, 2024; UNESCO, 2024). Given the urgency of climate change and the individual, societal, and ecological significance of climate literacy, the United Nations Educational, Scientific and Cultural Organization (UNESCO) advocates for the integration of climate change issues into school curricula to foster students’ climate literacy (UNESCO, 2023). Among various school subjects, science education has unique value in shaping students’ climate literacy, as climate literacy must be rooted in a solid scientific foundation (Choi et al., 2021). Climate change is a complex socio-scientific issue (Choi et al., 2021); mitigating and adapting to it requires not only scientific understanding and serious attitudes towards the climate problem but also the coordination of various stakeholders based on this foresight to foster collective action. However, due to a lack of scientific literacy and the inappropriate spread of pseudoscience, climate change skepticism has become rampant, hindering societal progress towards climate action (Sharma, 2012; Dunlap, 2013). This implies that while advocating for widespread and informed climate action, we must also publicly challenge climate change skepticism. Winning this debate relies on rigorous reasoning based on scientific evidence regarding climate change. This is precisely the strength of science education. It can ground the cultivation of students’ climate literacy in the development of their socio-scientific reasoning skills, allowing the two to mutually reinforce each other. Specifically, climate change provides a vivid topic and context for students’ socio-scientific reasoning. The complex thinking, comprehensive perspectives, continuous inquiry, and critical spirit emphasized by socio-scientific reasoning (Romine et al., 2020), when combined with climate change instruction, will deepen students’ understanding of the complexity of climate issues and enable them to make responsible decisions and informed actions based on synthesizing diverse existing perspectives and attitudes towards climate problems. Therefore, the relationship between science education and students’ climate literacy is receiving increasing research attention.

Research demonstrates that science education exerts multifaceted positive effects on students’ climate literacy. First, multiple studies utilizing pre- and post-standardized tests have confirmed a significant enhancement in students’ understanding of core climate change concepts following a period of science education (McNeill and Vaughn, 2012; Bodzin and Fu, 2014). For instance, the study by Bodzin and Fu (2014) revealed that after a 20-day geospatial curriculum, middle school students’ scores on assessments of atmosphere, greenhouse effect, and climate systems significantly increased from a pre-test average of 6.23 to a post-test average of 9.47, indicating that the science education intervention substantially strengthened students’ conceptual understanding of climate change. Second, several studies have found that systematically integrating climate change content into science curricula can effectively foster students’ positive attitudes and sense of responsibility towards climate issues (Kumar et al., 2023; Clark, 2024; Karpudewan et al., 2015). For example, Kumar et al. (2023), within the “Heat-Cool Initiative,” observed that secondary students participating in a thermal imaging curriculum not only showed significantly improved test scores but also underwent an attitudinal shift. They progressed from perceiving climate change as a distant and abstract threat to believing it is personally relevant and that they can effect positive change through concrete actions, such as opting for green infrastructure. Finally, science education also contributes to the transformation of students’ climate actions (Deisenrieder et al., 2020; Choi et al., 2021). Illustratively, Choi et al. (2021), through the implementation of an SSI-STEAM project, documented improvements in both the level of student engagement in climate actions and the breadth of action domains covered.

Although the aforementioned studies affirm the positive contribution of science education to cultivating students’ climate literacy, multiple empirical studies reveal significant imbalances in its effectiveness across cognitive, affective, and action dimensions. First, science education’s promotion of climate cognition has clear limitations. On one hand, the effectiveness of science curricula in promoting climate cognition shows strong topic dependence, often failing to reach significance levels in specific areas such as wetland conservation, solar activity, or adaptation strategies (Lombardi et al., 2013, 2018; Clark, 2024). On the other hand, even well-designed science instruction often struggles to systematically reconstruct students’ deeply ingrained pre-scientific conceptions, leading to persistent confusion and misconceptions about core mechanisms like the greenhouse effect (Porter et al., 2012; Bush et al., 2019). This unstable cognitive foundation weakens the translation of knowledge into affective identification. Research shows that science curricula, even when they lead to gains in climate knowledge, may not necessarily improve students’ attitudes towards the environment (Karpudewan et al., 2015) or personal concern about climate issues (Bush et al., 2019); even courses designed to strengthen beliefs and resilience have limited effects on alleviating psychological anxiety (Gupta et al., 2025). This barrier in translating cognition to attitude ultimately makes it difficult for science education to effectively promote behavioral change in students. Extensive evidence shows that mere mastery of scientific content is insufficient to support students’ willingness to act on complex climate challenges (Igboanugo and Naiho, 2024), nor does it drive students to take practical environmental actions (McNeill and Vaughn, 2012; Clark, 2024). Furthermore, the realization of individual agency remains constrained by social norms and infrastructure beyond the curriculum system, often causing the development of action to lag significantly behind the acquisition of knowledge (Gupta et al., 2025).

Given the divergent findings among existing individual studies, it is necessary to employ meta-analysis to synthesize existing research conclusions and holistically explore the impact of science education on students’ climate literacy. Current relevant meta-analyses have primarily investigated general factors influencing climate literacy (Bergquist et al., 2023) or assessed the overall impact of environmental education on environmental literacy (Van De Wetering et al., 2022). Although Aeschbach et al. (2025) recently published a meta-analysis on the effectiveness of climate change education (CCE), their aim was to evaluate the effects of CCE in a broad sense. The literature they analyzed thematically encompassed environmental education and education for sustainable development extensively, without a specific focus on cultivating students’ climate literacy. In terms of implementation, it included both thematic education and multidisciplinary integrated instruction, thereby obscuring the distinct contribution of science curricula to climate change education. Consequently, there is currently a lack of meta-analytic research specifically targeting the effectiveness of implementing climate change education within school science education—that is, on the efficacy of cultivating students’ climate literacy.

The present study aims to employ a meta-analysis to examine whether school science education serves as an effective vehicle for developing students’ climate literacy, thereby clarifying the unique contribution of science education to climate change education. Furthermore, it seeks to investigate the factors that influence the effectiveness of school science education in fostering students’ climate literacy, to identify the specific conditions under which science education can effectively fulfill this role. This original research is expected to provide an evidence-based foundation and practical insights for enhancing school science curricula integrated with climate change education and for improving civic climate literacy. To achieve these research objectives, the following four research questions are proposed:

*RQ1*: What is the estimated overall effect of school science education on students’ climate literacy under heterogeneous instructional, disciplinary, and contextual conditions?

*RQ2*: How does the impact of school science education differ across the cognitive, attitudinal, and behavioral dimensions of climate literacy?

*RQ3*: Under what disciplinary conditions does school science education exert stronger or weaker effects on students’ climate change?

*RQ4*: To what extent do instructional strategies, educational level, and intervention duration function as boundary conditions that moderate the relationship between school science education and students’ climate literacy?

## 2 Methods

This study strictly adheres to the PRISMA guidelines (Page et al., 2021). The research process primarily comprises the following six steps: (1) systematic literature search and selection, (2) data extraction and coding, (3) effect size calculation, (4) effect size pooling based on a three-level model, (5) moderator analysis, and (6) discussion of findings.

### 2.1 Literature search and screening

A systematic search was conducted across five academic databases: Web of Science, EBSCO, Scopus, Taylor & Francis Online, and ERIC, to identify experimental or quasi-experimental studies investigating the role of school science education in developing students’ climate literacy. The literature search included all publications available through May 2025. The search strategy employed two distinct sets of keywords. The first group of keywords was related to science education, including “science education,” “chemistry,” “biology,” “geography,” “Earth science,” and “physics.” The second set of keywords was related to climate literacy, including “climate literacy,” “climate change cognition,” “attitudes towards climate change,” and “climate action.” Boolean logic operators (AND, OR) were used to combine these two sets of keywords.

### 2.2 Inclusion and exclusion criteria

The study selection process adhered to the following pre-defined criteria:

1. Included studies must investigate the impact of school science education on students’ climate literacy. Consequently, studies that did not report outcomes relevant to climate literacy (e.g., climate change cognition, attitudes, or actions) were excluded.
2. This study focuses on general science education implemented within formal school education systems. The scope of inclusion covers several instructional contexts: (a) situations in which climate literacy is holistically and longitudinally integrated across disciplines such as science, physics, chemistry, biology, Earth science, and geography; (b) situations in which climate-related topics are embedded within a specific unit or chapter of a standard science curriculum; and (c) interdisciplinary natural science courses explicitly centered on climate change. To more precisely identify the unique contribution of school-based natural science education to climate change education, this study excludes climate change instruction delivered in humanities and social science courses (e.g., language arts and history) based on disciplinary characteristics. In addition, climate change education conducted in out-of-school, non-formal learning environments (e.g., summer camps and museum education) is excluded based on educational attributes. Instructional contexts that simultaneously involve natural sciences and humanities or that combine in-school and out-of-school settings are also excluded.
3. As for participants, with consideration that climate change education should span all educational stages, students from primary school, secondary school, and higher education within formal education systems are all included in this study. At the same time, recognizing potential developmental differences across educational stages, subgroup analyses by educational level are conducted to examine whether the effects are consistent across primary, secondary, and higher education students.
4. The study design was required to be experimental or quasi-experimental. Included studies must feature at least one control group or incorporate both pre-test and post-test measurements.
5. Sufficient quantitative data for calculating effect sizes (e.g., sample size, means, standard deviations) had to be reported in the study.
6. Publication language was restricted to English.

**Figure 1:**
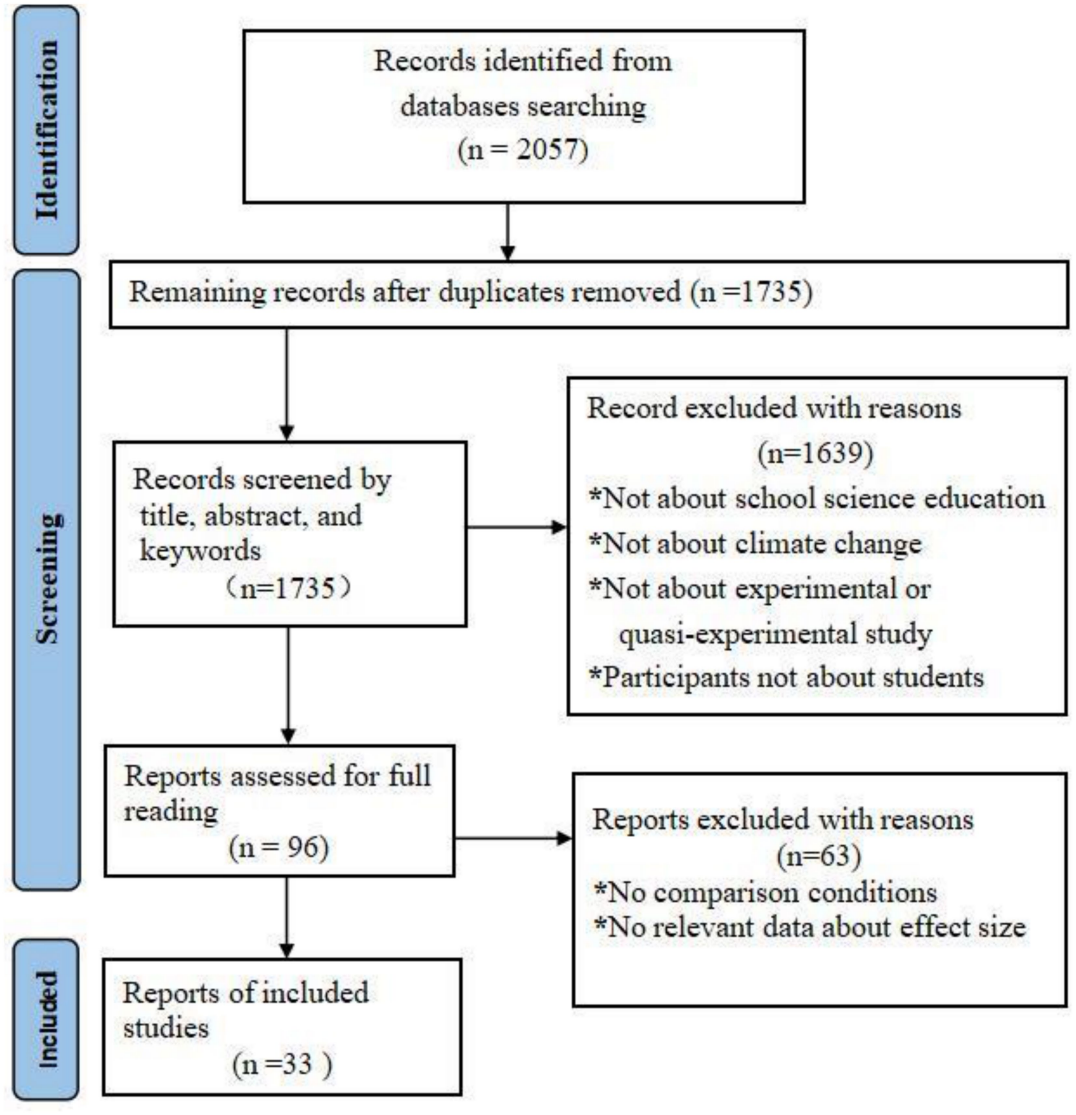
Selection flowchart based on PRISMA usage.

Figure 1 presents the literature screening process following PRISMA guidelines, comprising three core stages: identification, screening, and final inclusion. First, in the identification stage, 2057 studies were initially retrieved from databases. After removing 322 duplicate studies automatically and manually, 1735 studies remained for the screening stage. During screening, 1,639 studies were excluded by reading titles, abstracts, and keywords for the following specific reasons: (1) not related to school science education, (2) not related to climate change content, (3) not experimental/quasi-experimental design, (4) participants not students. Finally, the full texts of the remaining 96 studies were reviewed. Of these, 63 studies were excluded due to lacking a control condition or usable effect size data. Ultimately, 33 studies were included in the analysis, providing 61 effect sizes suitable for meta-analysis (some studies provided multiple effect sizes due to containing multiple analysis indicators).

### 2.3 Data coding

During the data extraction and coding phase, we systematically organized and meticulously recorded information from the included literature according to a predefined coding scheme, as detailed in Table 1. The process involved three key stages. First, basic study characteristics were extracted, including the author names, publication year, sample size, and the country where the study was conducted. Second, we conducted an in-depth analysis of the primary content of each study. This involved coding the outcome variables (namely, climate literacy and its dimensions: climate change knowledge, attitudes towards climate change, and climate action) alongside potential moderator variables that could influence the effectiveness of school science education in cultivating student climate literacy. Finally, numerical data necessary for calculating effect sizes were extracted. To ensure coding reliability, the first two researchers independently extracted features and coded the included literature. Statistical results showed excellent initial inter-rater reliability (Cohen’s *κ* = 0.951). Minor disagreements were resolved through discussion to reach full consensus.

**Table 1:** Literature coding table.

Author (year)	*N*	State	OV	Discipline	IS	EI	ID
Aksit et al. (2018)	122	USA	Cognition	Earth science	W/OD-T	HE	1–6 months
Aksit et al. (2018)	122	USA	Attitude	Earth science	W/OD-T	HE	1–6 months
Svihla and Linn (2012)	134	USA	Cognition	Earth science	W/D-T	SE	1–6 months
Svihla and Linn (2012)	238	USA	Cognition	Earth science	W/D-T	SE	1–6 months
Clark (2024)	25	USA	Cognition	Chemistry	W/OD-T	SE	> 6 months
Lombardi et al. (2018)	153	USA	Cognition	Earth science	W/OD-T	SE	> 6 months
Lombardi et al. (2018)	187	USA	Cognition	Earth science	W/OD-T	SE	> 6 months
McNeill and Vaughn (2012)	75	USA	Cognition	Biology	W/OD-T	SE	> 6 months
McNeill and Vaughn (2012)	22	USA	Cognition	Biology	W/OD-T	SE	> 6 months
Liu et al. (2022)	158	USA	Cognition	Chemistry	W/D-T	HE	> 6 months
Mostacedo-Marasovic et al. (2023)	94	USA	Cognition	Earth science	W/D-T	SE	> 6 months
Mostacedo-Marasovic et al. (2023)	53	USA	Cognition	Earth science	W/D-T	SE	> 6 months
Mostacedo-Marasovic et al. (2023)	269	USA	Attitude	Earth science	W/D-T	SE	> 6 months
Dawson and Carson (2020)	30	Australia	Cognition	Science	W/OD-T	SE	< 1 month
Reinfried et al. (2012)	122	Switzerland	Cognition	Geography	W/OD-T	SE	< 1 month
Reinfried et al. (2012)	128	Switzerland	Cognition	Geography	W/OD-T	SE	< 1 month
Porter et al. (2012)	66	Canada	Cognition	Science	W/OD-T	SE	< 1 month
Porter et al. (2012)	66	Canada	Cognition	Science	W/OD-T	SE	< 1 month
Porter et al. (2012)	66	Canada	Cognition	Science	W/OD-T	SE	< 1 month
Igboanugo and Naiho (2024)	375	Nigeria	Attitude	Chemistry	W/OD-T	SE	> 6 months
Igboanugo and Naiho (2024)	350	Nigeria	Attitude	Chemistry	W/OD-T	SE	> 6 months
Igboanugo and Naiho (2024)	375	Nigeria	Cognition	Chemistry	W/OD-T	SE	> 6 months
Igboanugo and Naiho (2024)	350	Nigeria	Cognition	Chemistry	W/OD-T	SE	> 6 months
Drewes et al. (2018)	42	USA	Cognition	Earth science	W/OD-T	SE	< 1 month
Bodzin and Fu (2014)	956	USA	Cognition	Geography	W/D-T	SE	< 1 month
Lombardi et al. (2013)	86	USA	Cognition	Earth science	W/OD-T	SE	> 6 months
Lombardi et al. (2013)	83	USA	Cognition	Earth science	W/OD-T	SE	> 6 months
Eggert et al. (2017)	96	Germany	Cognition	Science	W/D-T	SE	< 1 month
Eggert et al. (2017)	93	Germany	Cognition	Science	W/D-T	SE	< 1 month
Choi et al. (2021)	31	Korea	Cognition	Science	W/OD-T	SE	< 1 month
Choi et al. (2021)	31	Korea	Attitude	Science	W/OD-T	SE	< 1 month
Choi et al. (2021)	31	Korea	Action	Science	W/OD-T	SE	< 1 month
Karpudewan et al. (2015)	60	Malaysia	Cognition	Science	W/OD-T	PE	1–6 months
Karpudewan et al. (2015)	60	Malaysia	Attitude	Science	W/OD-T	PE	1–6 months
Bhattacharya et al. (2021)	70	USA	Cognition	Earth science	W/D-T	SE	< 1 month
Cox et al. (2014)	18	USA	Cognition	Geography	W/D-T	HE	> 6 months
Cox et al. (2014)	22	USA	Attitude	Geography	W/D-T	HE	> 6 months
Varma and Linn (2012)	190	USA	Cognition	Science	W/D-T	SE	< 1 month
Bush et al. (2019)	39	Canada	Cognition	Earth science	W/D-T	SE	1–6 months
Bush et al. (2019)	40	Canada	Cognition	Earth science	W/D-T	SE	1–6 months
Zangori et al. (2017)	50	USA	Cognition	Biology	W/D-T	SE	< 1 month
Chang et al. (2018)	30	Singapore	Cognition	Geography	W/OD-T	SE	< 1 month
Ozyazici and Ceyhan (2025)	62	USA	Cognition	Science	W/D-T	HE	< 1 month
Harker-Schuch et al. (2020)	401	Austria& Australia	Cognition	Science	W/D-T	PE	< 1 month
Holthuis et al. (2014)	742	USA	Cognition	Science	W/OD-T	SE	1–6 months
Karpudewan and Khan (2017)	30	Malaysia	Cognition	Biology	W/D-T	SE	1–6 months
Karpudewan and Khan (2017)	32	Malaysia	Cognition	Biology	W/OD-T	SE	1–6 months
Cartwright et al. (2021)	47	USA	Cognition	Earth science	W/OD-T	SE	< 1 month
Lester et al. (2006)	420	USA	Cognition	Earth science	W/OD-T	PE	> 6 months
Smith et al. (2019)	511	USA	Cognition	Science	W/D-T	SE	> 6 months
Nussbaum et al. (2015)	59	USA	Cognition	Earth science	W/D-T	SE	< 1 month
Nussbaum et al. (2015)	61	USA	Cognition	Earth science	W/D-T	SE	< 1 month
Nussbaum et al. (2015)	61	USA	Attitude	Earth science	W/D-T	SE	< 1 month
Nussbaum et al. (2015)	60	USA	Attitude	Earth science	W/D-T	SE	< 1 month
Markowitz et al. (2018)	19	USA	Cognition	Biology	W/D-T	SE	1–6 months
Petersen et al. (2020)	52	Denmark	Attitude	Science	W/D-T	SE	< 1 month
Petersen et al. (2020)	52	Denmark	Action	Science	W/D-T	SE	< 1 month
Petersen et al. (2020)	52	Denmark	Cognition	Science	W/D-T	SE	< 1 month
Petersen et al. (2020)	50	Denmark	Attitude	Science	W/D-T	SE	< 1 month
Petersen et al. (2020)	50	Denmark	Action	Science	W/D-T	SE	< 1 month
Petersen et al. (2020)	50	Denmark	Cognition	Science	W/D-T	SE	< 1 month
	8,419						

*N*, total sample size; OV, Outcome variable; IS, Instruction strategy; W/D-T, with digital technology support; W/OD-T, without digital technology support; EI, educational level; PE, primary education; SE, secondary education; HE, higher education; ID, intervention duration.

This study used the Medical Education Research Study Quality Instrument (MERSQI) to assess the methodological quality of the included empirical studies (Reed et al., 2007). This tool quantifies study quality across six domains encompassing 10 items (total score 18), with an average score above 9 indicating sufficient quality (Smith and Learman, 2017). The MERSQI quality assessment score was 14.77 (SD = 0.82), indicating that the studies included in this meta-analysis were of very good quality.

#### 2.3.1 Outcome variables

Definitions and measurements of climate literacy vary across existing scholarship. Different researchers have conceptualized and assessed this construct through distinct dimensional frameworks. Some studies have operationalized climate literacy through knowledge, perception, and action (Choi et al., 2021); others have employed knowledge, values, attitudes, and action intentions (Tolppanen et al., 2022); while another approach utilizes knowledge, attitudes, climate-friendly behaviors, personal concern, and influence on others (Kuthe et al., 2019). This study divides climate literacy into three sub-dimensions: cognition, attitude, and action. This division not only aligns with the aforementioned literature on climate literacy but also broadly corresponds to UNESCO’s consideration of learning objectives for sustainable development goals divided into cognitive, socio-emotional, and behavioral dimensions (UNESCO, 2017), and it highly matches the actual situation of the studies included in this meta-analysis. It should be noted that “attitude” is used instead of “affect/emotion” because “attitude” is a more encompassing term that can cover emotions, attitudes, and values (Eagly and Chaiken, 1993; Fishbein and Ajzen, 1977). “Action” is used instead of “behavior” because Sustainable Development Goal 13 is “Climate Action” (UN, 2015). In this study, climate change cognition refers to students’ systematic understanding of fundamental facts, scientific principles (e.g., the greenhouse effect), causes, and consequences of climate change. Attitudes toward climate change encompass students’ emotional tendencies, degree of concern, sense of responsibility, and willingness to respond to the issue. Climate actions constitute the practical steps students take to mitigate and adapt to climate change.

#### 2.3.2 Moderator variables

To explore sources of between-study heterogeneity, and drawing on prior meta-analyses, literature reviews, and empirical studies, as well as the shared characteristics of the included studies, four categories of moderator variables were selected for analysis: discipline, instructional strategy, educational level, and intervention duration.

##### 2.3.2.1 Discipline

Discipline was selected as a moderator variable for two main reasons. First, school science education encompasses multiple disciplines, and failing to analyze specific subjects may obscure disciplinary differences. Second, prior research has suggested that different disciplines may vary in their effectiveness in promoting students’ climate literacy. For example, a systematic review by Nepraš et al. (2022) found that geospatial curricula significantly enhanced students’ climate change knowledge. An empirical study by Onuoha et al. (2021) involving secondary school students showed that geography had a stronger effect on promoting climate awareness than chemistry, biology, and agricultural science. Empirical studies of university students by Powers et al. (2021) and Kannangara et al. (2025) also reported significant differences in climate literacy levels across disciplines. Based on this evidence, it is well justified to treat discipline as a potential moderator of the effectiveness of science education in fostering students’ climate literacy. Consistent with the disciplines represented in the included studies, this research examines five categories: chemistry, biology, geography, general science, and Earth science.

##### 2.3.2.2 Instructional strategy

Instructional strategy was selected as a moderator because climate literacy can be cultivated through a wide range of pedagogical approaches. Conducting subgroup analyses on instructional strategies allows for a more nuanced understanding of the effectiveness of different approaches and informs the design of more effective interventions. Given the high diversity of instructional strategies used in the included studies—ranging from digital approaches such as computer simulations and online platforms to non-digital approaches such as fieldwork and classroom lectures—examining each strategy separately without classification would result in an insufficient number of studies per subgroup, severely reducing statistical power (Hillmayr et al., 2020). Taking into account the characteristics of the included studies (particularly how different classifications affect the robustness of the analysis), trends in the digitalization of contemporary education, and common categorizations in the literature, this study adopts a binary classification of “with digital technology support” and “without digital technology support” to examine the role of digital technology in enhancing students’ climate literacy through science education.

On the one hand, this digital versus non-digital distinction is well supported by previous research. Binary comparisons between technology-integrated instruction and traditional instruction are common in meta-analyses of science education. Bayraktar (2001), in examining the effectiveness of computer-assisted instruction in science education, compared computer-assisted and traditional instruction and argued that the most fundamental and critical analysis for establishing the value of technology lies in an overall comparison between technology-integrated classrooms and traditional classrooms. Similarly, Hillmayr et al. (2020) emphasized in their meta-analysis of mathematics and science education that, given the diversity and fragmentation of research on digital tools, comparing digital tools as a whole with traditional instruction is currently the most appropriate approach for evaluating the overall effectiveness of technology in STEM education. This classification logic is also supported by empirical research in climate change education. For example, Karpudewan et al. (2015), in a study of climate literacy development among Malaysian elementary students, contrasted digital inquiry activities supported by presentations and simulation games with traditional lecture-based instruction relying on blackboard drawings and textbooks, demonstrating the applicability of this categorization in climate education contexts. On the other hand, prior studies have reported inconsistent findings regarding the effectiveness of digital technologies, underscoring the importance of conducting such subgroup analyses in the present study. Abbey et al. (2024) noted that although educational technology has a small positive effect on student learning outcomes, this effect varies substantially across contexts and modes of implementation. Fike et al. (2016) found through empirical analysis that traditional instructional methods were more effective than modern educational technologies in helping students complete coursework and improve academic performance. In contrast, a systematic review by Hajj-Hassan et al. (2024) concluded that the use of digital tools is more effective in increasing students’ attention to climate change and promoting pro-environmental behaviors.

##### 2.3.2.3 Educational level

Educational level was selected as a moderator because the study includes student samples from multiple educational stages. Given potential developmental differences across stages, subgroup analyses are necessary to examine whether the effects are consistent among elementary, secondary, and higher education students. Previous research has also identified educational level as an important factor influencing environmental-related literacies. Gökmen (2021), through a meta-analysis, found that educational level significantly moderated students’ environmental attitudes. Numerous studies have shown that educational attainment is an important determinant of climate literacy, with higher educational levels associated with more positive climate-related cognition, behaviors, and support for climate policies (Leiserowitz et al., 2021). Bruine de Bruin and Dugan (2022) further noted that high school and college students, due to their more systematic accumulation of knowledge, are more likely than elementary school students to express concern about climate change. In contrast, a meta-analysis by Świątkowski et al. (2024) on interventions promoting children’s pro-environmental behaviors found that intervention effects declined with age. Accordingly, educational level was included as a moderator and categorized as primary education, secondary education, and higher education based on the included studies.

##### 2.3.2.4 Intervention duration

Intervention duration was selected as a moderator because climate change education conducted within formal science education systems is often conceptualized as an intervention with a defined time span. Understanding how long an intervention needs to be to produce effects—and whether longer interventions are more effective—can inform the design of future programs. Moreover, prior research has yielded inconsistent conclusions regarding the role of intervention duration. For example, meta-analyses by Aeschbach et al. (2025) and Çalik and Wiyarsi (2025) identified intervention duration as a significant moderator, whereas meta-analyses by Van De Wetering et al. (2022) and Doğan et al. (2023) found that duration did not significantly moderate the outcomes of environmental education or science education, highlighting the necessity of this subgroup analysis. Following Ardoin et al.’s (2018) systematic review of environmental education programs, which categorized program duration into three typical formats—single instructional units, semester-long activities, and academic-year implementations—and considering the natural cycles of school instruction (with typical instructional units lasting no more than 1 month and typical semesters spanning 1–6 months), intervention duration was categorized into three groups based on the actual conditions of the included studies and instructional practices: “< 1 month,” “1–6 months,” and “> 6 months.”

### 2.4 Data analysis

We first used an Excel spreadsheet to organize and extract the raw data needed to calculate effect sizes, including the sample sizes (*N*), means (M), and standard deviations (SD) for the experimental and control groups. Subsequent statistical analyses were performed in the R statistical environment (Version 4.3.1) using the metafor package. When calculating effect sizes, Hedges’g was chosen over Cohen’s d because Hedges’g more effectively reduces bias and provides a more precise effect size (Borenstein et al., 2009, p. 83–84; Güler et al., 2022).

Given that some included studies contained multiple related outcome variables, resulting in effect sizes nested within studies and violating the independence assumption of traditional meta-analysis, this study employed a three-level random-effects model based on the methodological frameworks of Cheung (2015) and Van den Noortgate et al. (2013) to effectively handle data dependency and correct for underestimated standard errors. This model used restricted maximum likelihood (REML) to estimate parameters, decomposing total variance into three levels: sampling error (Level 1), within-study variation (Level 2), and between-study variation (Level 3), to achieve precise estimation of heterogeneity sources.

For heterogeneity assessment, this study calculated the *Q* statistic and the proportion of variance components at each level to determine the sources and degree of heterogeneity. For moderator analysis, this study incorporated the preset moderator variables within the three-level model framework and used the Omnibus *F*-test to assess the overall significance of the moderators (Borenstein et al., 2009; Higgins et al., 2003).

Finally, this study used multiple methods to comprehensively assess publication bias: first, visual inspection via a funnel plot; second, statistical verification using Egger’s linear regression test (Egger et al., 1997). Regardless of the presence of publication bias, this study used the Trim-and-Fill Method (Duval and Tweedie, 2000) as a sensitivity analysis to assess the impact of potentially missing studies on the overall effect and to verify the robustness of the conclusions by comparing the pooled effect sizes before and after correction.

## 3 Results

### 3.1 Sensitivity analysis

To ensure the robustness of the analysis results, this study employed two methods for outlier diagnosis. The first was Cook’s distance diagnostic method (Viechtbauer and Cheung, 2010) to identify studies exerting excessive influence on the pooled effect size estimate. The second was the standardized residual (*z*-value) test method, defining effect sizes with absolute *z*-values >3.29 as outliers (Assink and Wibbelink, 2016). Diagnostic results showed that one study (Igboanugo and Naiho, 2024) had a very high *z*-value of 6.87 (*p* < 0.001), and its Cook’s distance was significantly higher than other studies. Since extreme outliers may increase residual heterogeneity and distort pooled effect size estimates (Viechtbauer and Cheung, 2010), this study removed this outlier (Igboanugo and Naiho, 2024, *g* = 16.39). Subsequent analyses were based on the corrected dataset (*k* = 60).

To further test the robustness of the corrected results, this study conducted a sensitivity analysis using the leave-one-out method, sequentially removing each study from the corrected sample and recalculating the pooled effect size. Results showed that regardless of which study was removed, the pooled effect size consistently remained within the range of 0.93–1.02, indicating that the overall effect was not unduly influenced by any single study.

### 3.2 The overall effect of school science education on students’ climate literacy

**Figure 2:**
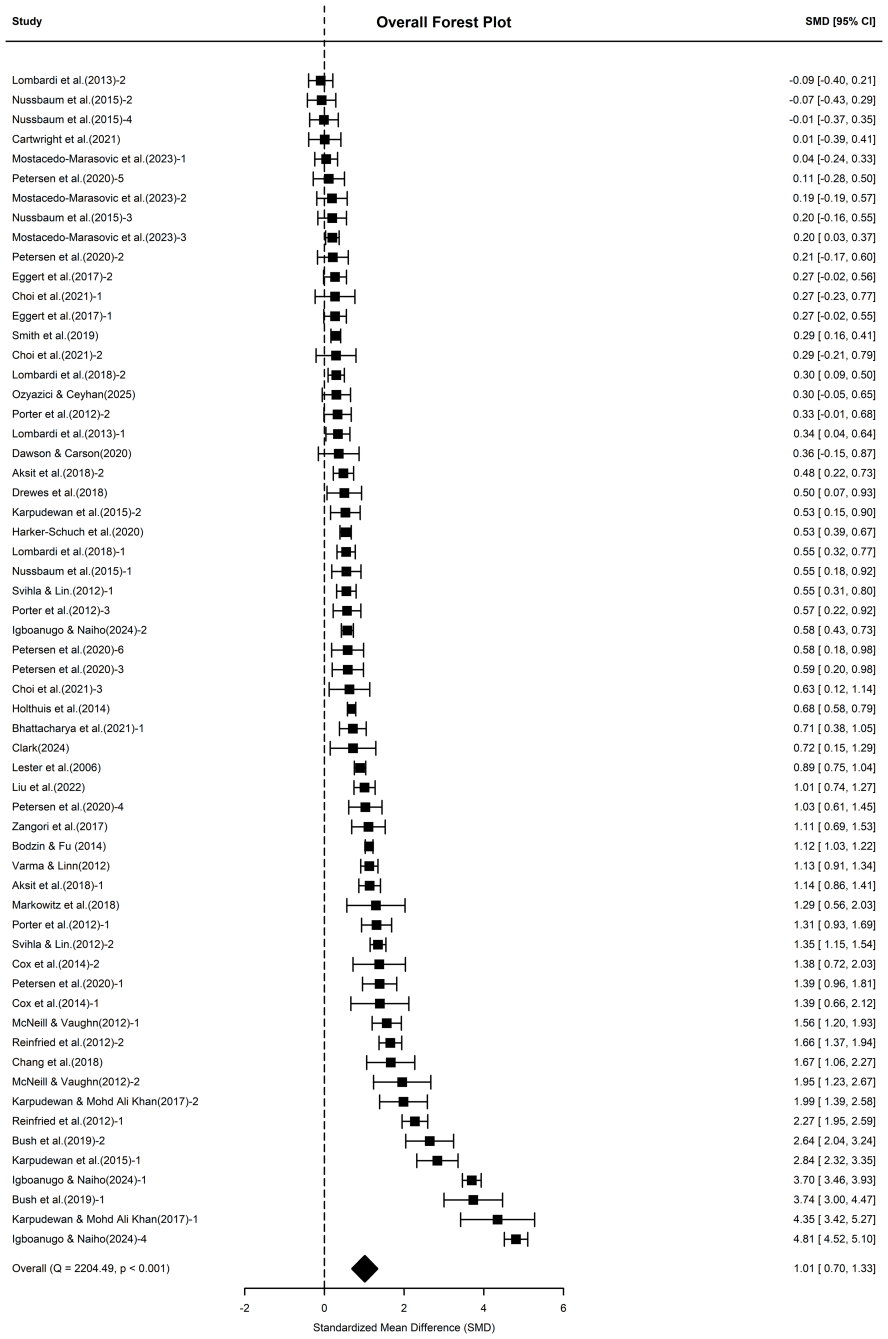
Forest plot of the overall effect.

This study included 33 studies (*N* = 8,044) from five continents, yielding 60 effect sizes (the forest plot is shown in Figure 2). These studies were primarily concentrated in North America (USA, *k* = 32; Canada, *k* = 5), followed by Europe (Germany, *k* = 2; Switzerland, *k* = 2; Denmark, *k* = 6; Austria, *k* = 1), then Asia (Korea, *k* = 3; Malaysia, *k* = 4; Singapore, *k* = 1), Africa (Nigeria, *k* = 3), and Oceania (Australia, *k* = 2). Overall, school science education had a significantly positive impact on students’ climate literacy (*g* = 1.01, 95% CI [0.70, 1.33], *p* < 0.001). According to Cohen (1988) standards, this represents a large effect. Aligning with the field’s meta-analysis effect size benchmarks, Aeschbach et al. (2025) explicitly defined *g* = 0.77 (climate knowledge) as a medium-to-large effect in their meta-analysis on climate change education, and Van de Wetering et al. (2022) defined an effect size of *g* = 0.95 (environmental knowledge) as a large effect in their environmental education meta-analysis. Therefore, from an educational perspective, this also demonstrates the strong effectiveness of school science education in enhancing students’ climate literacy.

This study conducted systematic heterogeneity analysis through Cochran’s *Q* test and three-level variance component decomposition, comparing robustness results after outlier removal (*k* = 60) with those including the outlier (*k* = 61) (see Table 2). In the three-level meta-analysis model, the heterogeneity test indicated high heterogeneity among the included studies [*Q*(59) = 2204.49, *p* < 0.001]. The three-level variance component decomposition revealed that the vast majority of variation stemmed from within-study variation (Level 2: *σ*^2^ = 0.55, 53.3%) and between-study variation (Level 3: *σ*^2^ = 0.48, 45.0%), rather than sampling error (Level 1 accounted for only 1.7%). Therefore, subsequent moderator variable analysis was needed to further explore the sources of heterogeneity.

**Table 2:** Three-level meta-analytic results: overall effect sizes and variance distribution.

Analysis type	*n*	*k* (*N*)	*g*	SE	95% CI	*Q* total	*σ* ^2^_level 2_	% var. level 2	*σ* ^2^_level 3_	% var. level 3
Without outliers	33	60 (8044)	1.01	0.16	[0.70, 1.33]	2204.49^***^	0.55	53.3%	0.48	45.0%
With outliers	33	61 (8419)	1.21	0.31	[0.61, 1.81]	3515.00^***^	3.64	79.7%	0.91	19.9%

*n*, number of studies; *k*, number of effect sizes; *N*, total sample size; *g*, Hedges’ *g* effect size; SE, standard error; CI, confidence interval; Q, heterogeneity value; *σ*^2^_level 2_, within-study variance; *σ*^2^_level 3_, between-study variance; % Var, percentage of variance; level 2, variance between effect sizes from the same study; level 3, variance between studies. ^*^p < 0.05; ^**^p < 0.01; ^***^p < 0.001.

### 3.3 The impact of school science education on student climate literacy across cognitive, attitudinal, and action dimensions

**Figure 3:**
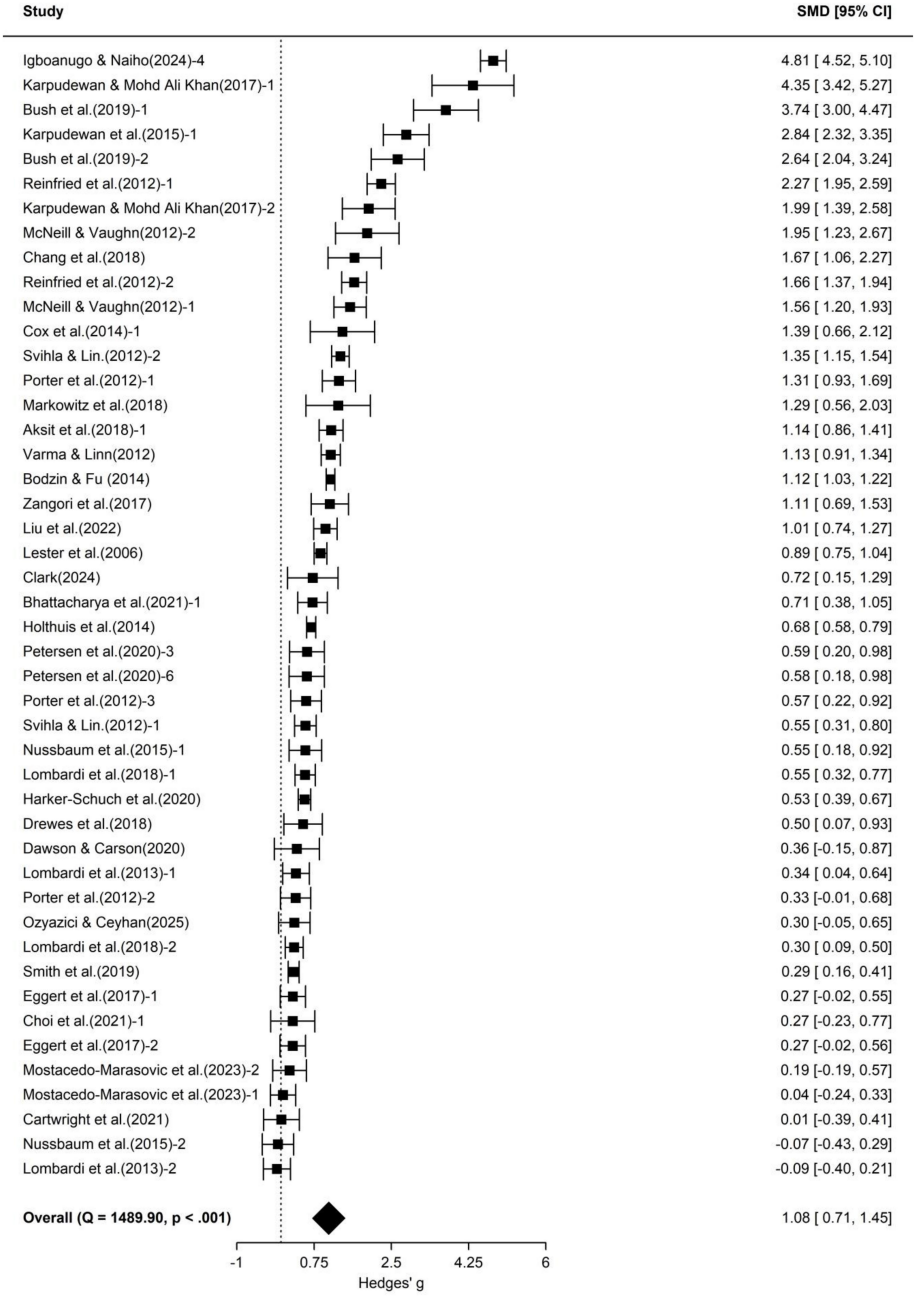
Forest plot of cognitive outcomes.

**Figure 4:**
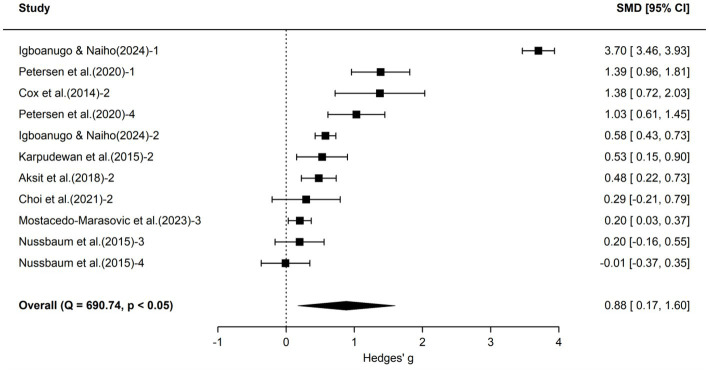
Forest plot of attitude outcomes.

**Figure 5:**
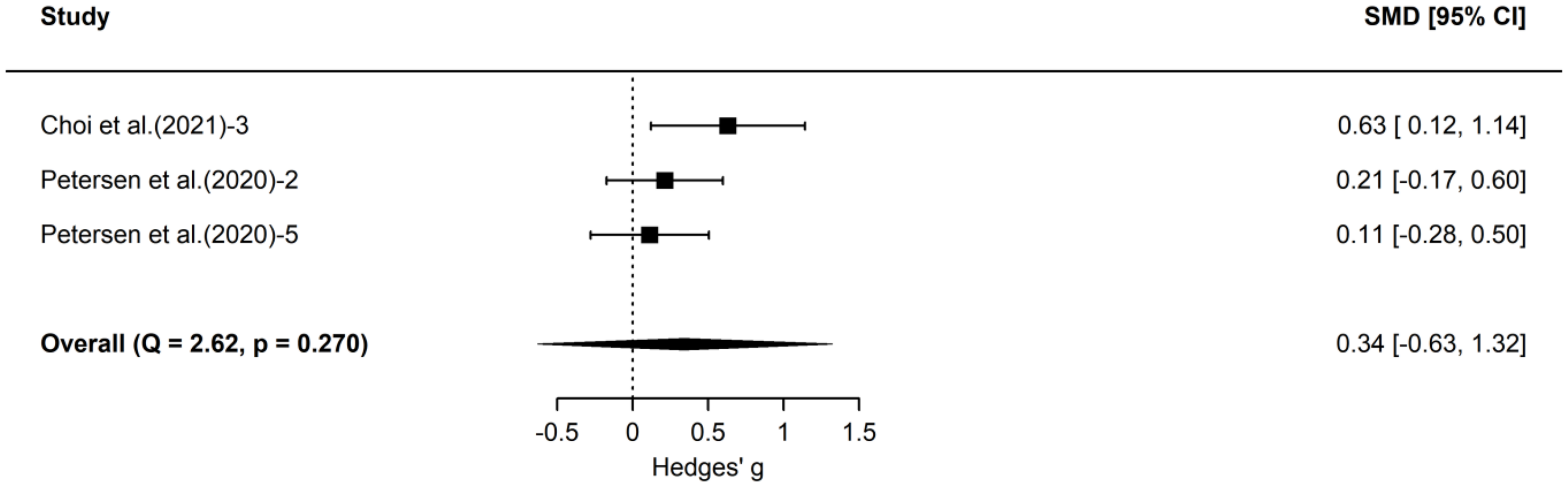
Forest plot of action outcomes.

To further explore the impact of school science education on different aspects of students’ climate literacy, we categorized the included 60 effect sizes into three dimensions—cognition, attitude, and action—and conducted separate three-level meta-analyses for each. Forest plots for each dimension are shown in Figures 3–5, and detailed results are presented in Table 3.

**Table 3:** Three-level meta-analysis results across three dimensions of climate literacy.

Dimension	*n*	*k* (*N*)	*g*	SE	95% CI	*Q* total	*σ* ^2^_level2_	% var. level 2	*σ* ^2^_level3_	% var. level 3
Cognition	33	46 (6457)	1.08	0.18	[0.71, 1.45]	1489.90^***^	0.19	17.2%	0.90	81.3%
Attitude	8	11 (1452)	0.88	0.32	[0.17, 1.60]	690.74^*^	1.07	96.8%	0.01	1.3%
Action	2	3 (135)	0.34	0.23	[−0.63, 1.32]	2.62	0.00	0.0%	0.07	58.1%

*n*, number of studies; *k*, number of effect sizes; *N*, total sample size; *g*, Hedges’ *g* effect size; SE, standard error; CI, confidence interval; Q, heterogeneity value; *σ*^2^_level 2_, within-study variance; *σ*^2^_level 3_, between-study variance; % Var, percentage of variance; level 2, variance between effect sizes from the same study; level 3, variance between studies. ^*^p < 0.05; ^**^p < 0.01; ^***^p < 0.001.

According to Table 3, the 46 effect sizes in the cognition dimension yielded an average effect size of 1.08 (95% CI [0.71, 1.45], *p* < 0.001), indicating that school science education significantly enhanced students’ climate change cognition levels. The 11 effect sizes in the attitude dimension yielded an average effect size of 0.88 (95% CI [0.17, 1.60], *p* < 0.05), indicating that school science education also had a significantly positive effect on students’ climate change attitudes. The action dimension contained only 3 effect sizes, with an average effect size of 0.34 (95% CI [−0.63, 1.32], *p* > 0.05), which did not reach statistical significance. Due to the very limited number of independent pieces of evidence available for this dimension, statistical power was insufficient, and the confidence interval was wide and included zero. The current analysis did not provide sufficient evidence to support an impact of school science education on students’ climate actions. Heterogeneity analysis indicated significant heterogeneity for both cognition and attitude. Although heterogeneity for the action dimension was not statistically significant, the three-level meta-analysis still revealed 58.1% between-study variance. However, after further subdivision into subgroups, the number of effect sizes in some subgroups became insufficient to support further exploration of heterogeneity sources with adequate statistical power. Therefore, this study did not conduct additional moderator variable analyses for the three sub-dimensions.

### 3.4 Moderator variables influencing the effectiveness of science education in developing students’ climate literacy

Given the high degree of heterogeneity, we conducted moderator variable analyses across four dimensions: educational level, discipline, intervention duration, and teaching strategy. Table 4 presents the results of the four moderator analyses under a three-level mixed-effects model.

**Table 4:** Results of the analysis of moderator variables.

Moderator variables	*n*	*k* (*N*)	*g*	SE	*p*	95% CI	*F* (*df*1, *df*2)	*σ* ^2^_level 2_	*σ* ^3^_level 3_
Discipline							*F* (4, 55) = 3.75^**^	0.58	0.23
Chemistry	3	5 (1258)	1.94	0.46	<0.001	[1.02, 2.87]			
Biology	4	6 (228)	1.92	0.42	<0.001	[1.08, 2.75]			
Geography	4	6 (1276)	1.57	0.41	<0.001	[0.75, 2.38]			
Earth science	11	21 (2440)	0.69	0.23	<0.01	[0.24, 1.15]			
Science	11	22 (2842)	0.65	0.23	<0.01	[0.19, 1.11]			
Instructional strategy							*F* (1, 58) = 0.00	0.56	0.49
With Digital Technology Support	17	31 (4185)	1.01	0.23	<0.001	[0.55,1.47]			
Without Digital Technology Support	17	29 (3859)	1.01	0.23	<0.001	[0.56, 1.47]			
Educational level							*F* (2, 57) = 0.04	0.56	0.51
Primary education	3	4 (941)	1.09	0.57	0.06	[−0.05, 2.22]			
Secondary education	26	50 (6599)	1.02	0.18	<0.001	[0.65, 1.39]			
Higher education	4	6 (504)	0.90	0.48	0.07	[−0.07, 1.87]			
Intervention duration							*F* (2, 57) = 3.13	0.56	0.37
1–6 months	7	12 (1638)	1.69	0.33	<0.001	[1.03, 2.35]			
> 6 months	10	18 (3251)	1.02	0.27	<0.001	[0.48, 1.57]			
< 1 month	16	30 (3155)	0.70	0.22	<0.01	[0.27, 1.14]			

*n*, number of studies; *k*, number of effect sizes; *N*, total sample size; *g*, Hedges’ *g* effect size; SE, standard error; CI, confidence interval; F (*df*1, *df*2), omnibus test of regression coefficient in the model; *p*, *p*-value of the omnibus test; *σ*^2^_level 2_, within-study variance; *σ*^2^_level 3_, between-study variance; ^*^p < 0.05; ^**^p < 0.01; ^***^p < 0.001.

#### 3.4.1 Discipline

Science education enhances students’ climate literacy through teaching in different disciplines. Among them, earth science (*k* = 21) and general science (*k* = 22) had more related studies, while biology (*k* = 6), geography (*k* = 6), and chemistry (*k* = 5) had relatively fewer studies. The between-group heterogeneity test indicated that the differences in effects between disciplines were significant [*F* (4, 55) = 3.75, *p* < 0.01], demonstrating that discipline significantly moderates the cultivation effect of school science education on students’ climate literacy. Specifically, the chemistry discipline produced the highest level of promoting effect on the effectiveness of school science education in developing students’ climate literacy (*g* = 1.94, *p* < 0.001). Biology (*g* = 1.92, p < 0.001) and geography (*g* = 1.57, *p* < 0.001) produced the next strongest effects. Earth science (*g* = 0.69, *p* < 0.01) and general science (*g* = 0.65, *p* < 0.01) had slightly weaker but still moderately significant promoting effects on cultivating students’ climate literacy through school science education. This finding stimulates further reflection on the relationship between disciplinary characteristics and climate literacy. However, the disciplinary ranking should be interpreted with caution, as imbalances in the number of included studies across disciplines (e.g., relatively few studies in chemistry, biology, and geography) may affect the stability of effect size estimates.

#### 3.4.2 Other moderating factors

##### 3.4.2.1 Instructional strategy

Teaching strategy primarily involved instruction with digital technology support (*k* = 31) and without digital technology support (*k* = 29). Statistical analysis results showed that both types of teaching strategies had a significantly positive impact on students’ climate literacy, with identical average effect sizes (*g* = 1.01, *p* < 0.001). Further between-group heterogeneity testing indicated that the difference in effects between the two teaching strategy types did not reach statistical significance [*F* (1, 58) = 0.00, *p* > 0.05]. This result indicates that regardless of whether digital technology is employed, there is no significant difference in the cultivation effect of school science education on students’ climate literacy; teaching strategy is not a key factor moderating this cultivation effect.

##### 3.4.2.2 Educational level

Among different educational levels, secondary education was the most concentrated area of research (*k* = 50), while higher education (*k* = 6) and primary education (*k* = 4) were relatively less researched areas. Statistical analysis results showed that the secondary education level produced a significantly positive effect (*g* = 1.02, *p* < 0.001). Although the effect sizes for primary education (*g* = 1.09, *p* > 0.05) and higher education (*g* = 0.90, *p* > 0.05) were substantial, they did not reach statistical significance. This may be attributable to the small number of included studies, resulting in insufficient statistical power rather than a true absence of effects. Further between-group heterogeneity testing indicated no significant differences in the effects of school science education on students’ climate literacy across educational levels [*F* (2, 57) = 0.04, *p* > 0.05]. Nevertheless, this trend warrants re-examination as future research accumulates more empirical evidence at the primary and higher education levels.

##### 3.4.2.3 Intervention duration

Regarding intervention duration, studies with “< 1 month” were the most numerous (*k* = 30), followed by “> 6 months” (*k* = 18) and “1 month-6 months” (*k* = 12). Statistical analysis results showed that all three intervention duration categories had significantly positive effects on students’ climate literacy: “1 month-6 months” (*g* = 1.69, *p* < 0.001), “> 6 months” (*g* = 1.02, *p* < 0.001), and “< 1 month” (*g* = 0.70, *p* < 0.01). However, no significant differences were found in the effects of school science education on students’ climate literacy across intervention durations (*F* (2, 57) = 3.13, *p* = 0.052), suggesting that intervention duration is not a key moderating factor.

### 3.5 Publication Bias test

**Figure 6:**
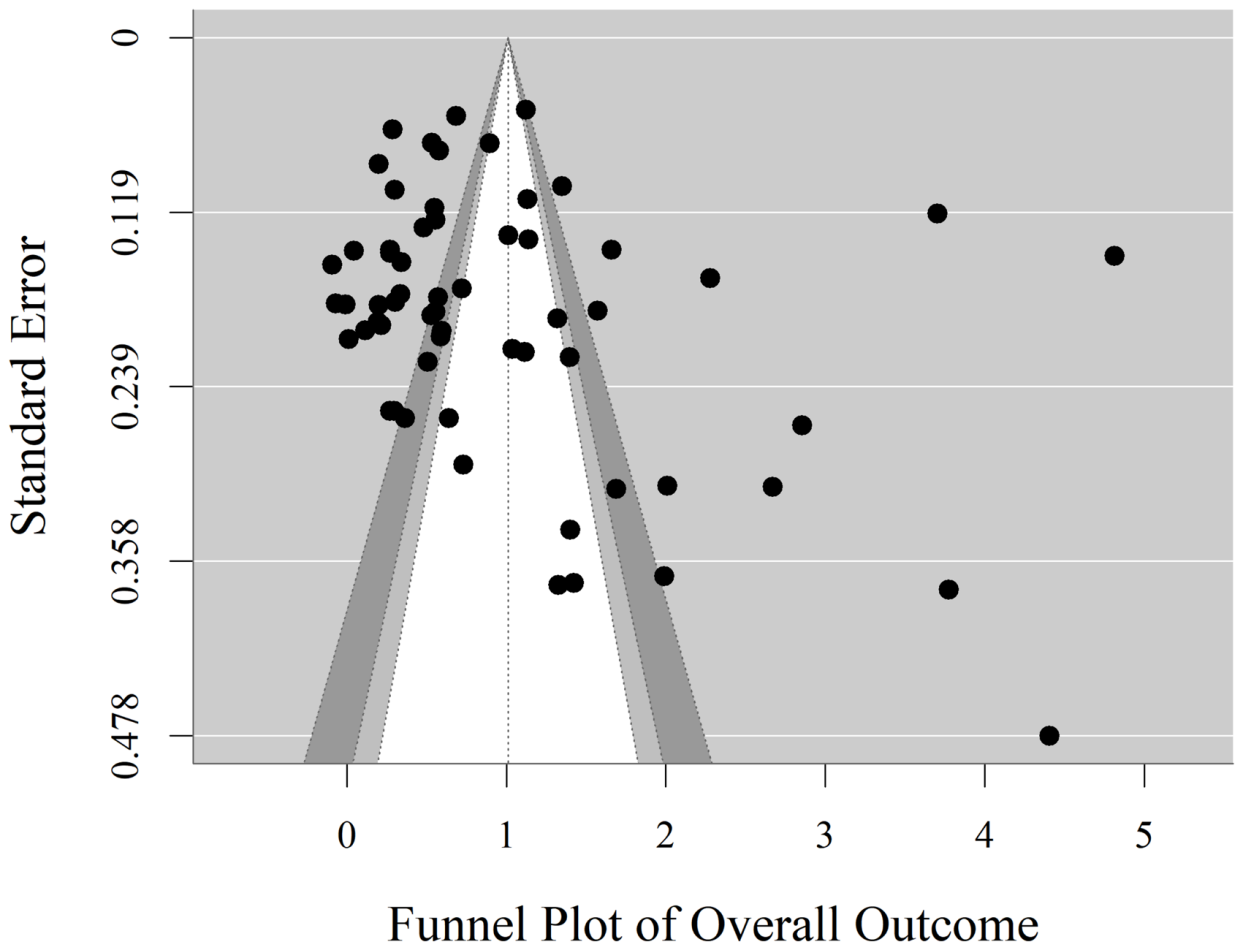
Funnel plot of overall outcomes.

Visual inspection of the funnel plot (Figure 6) showed an asymmetrical distribution of effect sizes. Egger’s regression test further statistically confirmed this asymmetry (*b* = 4.69, *z* = 3.22, *p* < 0.05), suggesting potential publication bias in this study. To ensure the robustness of the corrected results, this study conducted a publication bias correction analysis using the Trim-and-Fill Method (Duval and Tweedie, 2000). The analysis results indicated no identification of potentially missing studies; the overall effect size remained consistent before and after correction (*g* = 1.01). This result suggests that the asymmetry observed in Egger’s test is more likely due to high heterogeneity among studies rather than systematic publication bias, indicating good robustness of the research conclusions.

**Figure 7:**
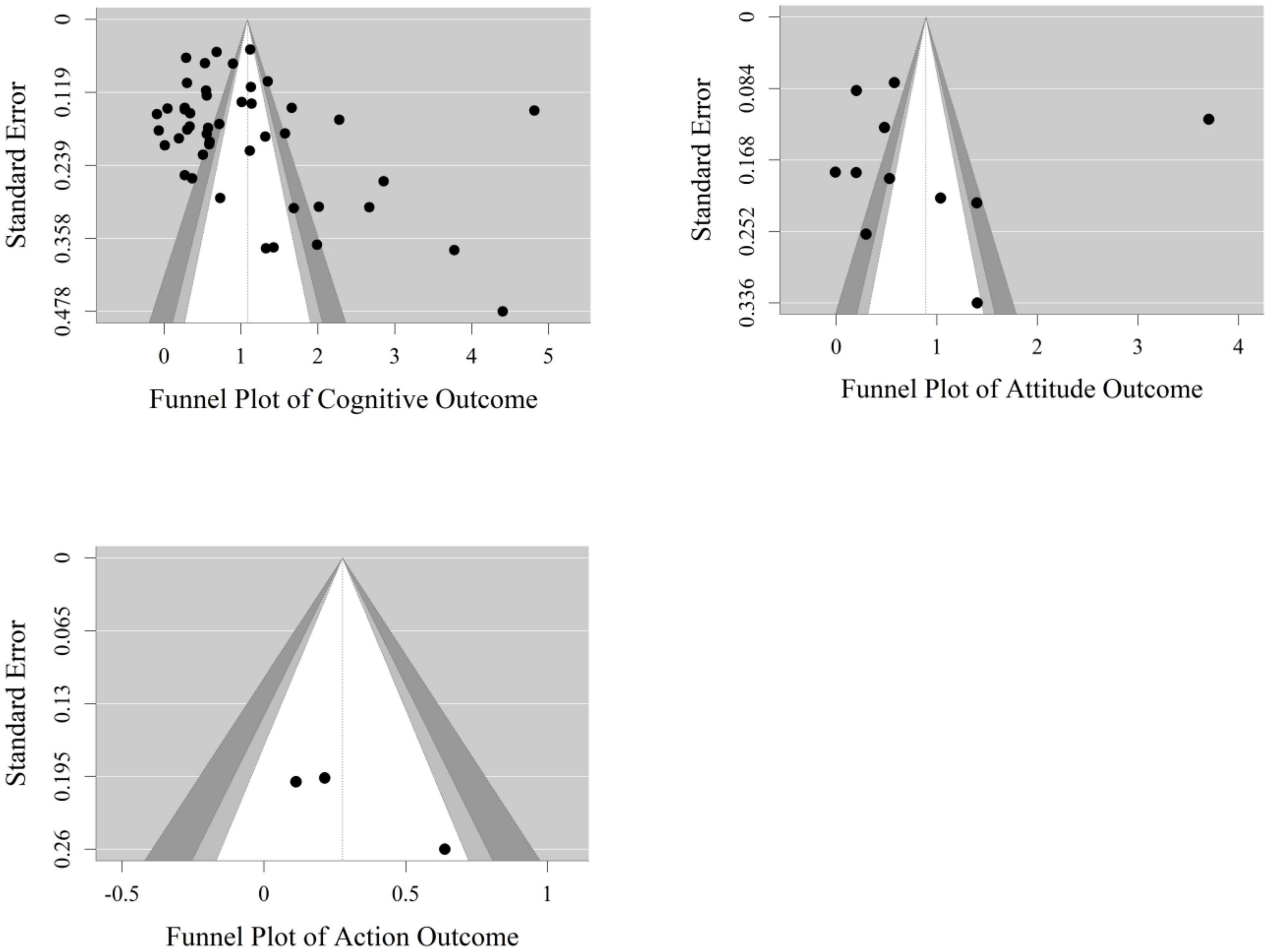
Funnel plots for cognitive, attitude, and action.

Separate publication bias tests were conducted for the cognition, attitude, and action dimensions. Visual inspection of the funnel plots (see Figure 7) showed that the effect size distributions for all three dimensions did not exhibit ideal symmetry, but the bias characteristics and statistical results differed across dimensions. For the cognition dimension, the funnel plot appeared asymmetric, and Egger’s regression test confirmed significant asymmetry (*b* = 5.50, *z* = 3.56, *p* < 0.05). However, the trim-and-fill analysis results showed no need to impute missing studies; the corrected overall effect size remained at *g* = 1.08. This indicates that the effect sizes in the cognition dimension were not substantially interfered with by publication bias. For the attitude dimension, Egger’s regression test found no significant evidence of bias (*b* = −0.05, *z* = −0.01, *p* = 0.99), indicating insufficient evidence to prove publication bias in the attitude dimension. For the action dimension, according to meta-analysis methodological norms (Assink and Wibbelink, 2016), when the number of included effect sizes is <10 (3 effect sizes for the action dimension), the statistical power of Egger’s test and the trim-and-fill method is insufficient to support reliable conclusions; therefore, further publication bias testing was not conducted.

## 4 Discussion

In reviewing previous literature, although the topic of school science education for cultivating students’ climate literacy has received extensive attention from numerous scholars, there is a lack of a comprehensive analysis of the effects of school science education in developing students’ climate literacy. To gain a more comprehensive understanding of the effects of school science education on students’ climate literacy, this study conducted a meta-analysis, which not only verified the positive effects of school science education on students’ climate literacy but also verified which factors affected this nurturing effect under different conditions.

The 60 effect sizes from the 33 included articles produced a significantly positive impact, confirming the effectiveness of school science education in enhancing students’ climate literacy. This result resonates with findings from multiple climate education meta-analyses (Aeschbach et al., 2025; Bottin et al., 2023; van de Wetering et al., 2022). Although previous studies either focused on the role of environmental education on environmental literacy, directly focused on climate change education, or discussed climate literacy in generalized educational contexts without specifically targeting the impact of school science education on students’ climate literacy, they all reached a consistent conclusion: providing students with relevant environmental or climate change education can have a significantly positive impact on their climate literacy. Simultaneously, this study found significant effect sizes for the cognition and attitude dimensions, with school science education being more effective in promoting students’ climate change cognition. The effect size for the action dimension did not reach significance. This further validates the common pattern in climate literacy cultivation of “cognition easy to improve, action difficult to translate” (Larrain et al., 2024; Aeschbach et al., 2025; Van de Wetering et al., 2022). It is important to note that since the action dimension only included 2 studies and 3 effect sizes, there may be issues of insufficient statistical power. This suggests that conclusions regarding the action dimension are highly exploratory and should be interpreted with caution.

This study found high heterogeneity among the included effect sizes. Through three-level variance decomposition, it was found that within-study variation (Level 2) accounted for 53.3% of the total, indicating that differences in literacy dimensions may be a core source of heterogeneity. In this study, the high effect size for school science education promoting climate literacy was primarily contributed by the cognition dimension. This aligns with the phenomenon noted by UNESCO that climate cognition learning receives widespread emphasis in teaching practice (UNESCO, 2019). Furthermore, SDG 4.7 lists “ensuring that all learners acquire the knowledge and skills needed to promote sustainable development” as a core global education task. Formal science education, with its structured, systematic curriculum framework, becomes a key vehicle for achieving this goal, capable of producing significant knowledge gains in a short time (Sánchez-Almodóvar et al., 2022). From a measurement perspective, climate change cognition (e.g., climate knowledge) has clear standardized assessment tools, and its improvement is easily quantifiable (Aeschbach et al., 2025; Bottin et al., 2023), which naturally manifests as significant effect sizes in meta-analysis. Moreover, current educational practice itself centers on knowledge transmission, with curriculum design, teaching implementation, and resource allocation tilting towards cognitive cultivation, further strengthening intervention effectiveness at this level (Aeschbach et al., 2025; Bottin et al., 2023). More importantly, the rigorous mechanistic deconstruction of climate issues by science education endows cognitive learning with irreplaceable depth. For example, chemistry disciplines analyze carbon cycle mechanisms from a molecular perspective, and biology disciplines interpret ecosystem feedback mechanisms to climate change (Mahaffy et al., 2024). This in-depth analysis of scientific principles allows students to establish rigorous causal logic, making improvements at the cognitive level appear particularly solid.

Through the three-level meta-analysis model, we found that 45% of the total heterogeneity also stemmed from between-group differences. Moderator effect analysis showed that discipline was a source of this heterogeneity, while teaching strategy, educational level, and intervention duration did not significantly influence the research results.

Our analysis confirmed that discipline significantly moderates the impact of school science education on students’ climate literacy, which aligns with Onuoha et al. (2021). However, a nuanced discrepancy exists in the ranking of disciplinary effects. Onuoha et al. (2021) identified geography as the most effective discipline for enhancing students’ climate awareness among the four disciplines examined, followed by chemistry, biology, and science. In contrast, our meta-analysis revealed that chemistry yielded the largest effect size, followed by biology, geography, Earth science, and science. This discrepancy may stem from methodological differences. Onuoha et al. (2021)’ study was a single empirical research in which the authors used correlation analysis to reveal associations between disciplines and climate awareness. Our study employed meta-analysis, drawing conclusions based on the synthesis of multiple empirical studies. The reason chemistry produced the most significant moderating effect may lie in its ability to explain the causes of climate change at the micro-mechanistic level (e.g., molecular structures of greenhouse gases), providing students with a solid conceptual knowledge base (Versprille and Towns, 2015). Simultaneously, its systematic exposition of material cycles (e.g., the carbon cycle) can link individual actions to global impacts, thereby effectively cultivating students’ systems thinking skills (Mahaffy et al., 2024). Furthermore, chemistry education can make abstract solutions concrete and credible by explaining the chemical principles behind teaching content such as carbon capture and new energy conversion, making students aware of the realistic possibility of human actions affecting the climate system, thus effectively promoting the transformation of cognition into action intention (Mahaffy et al., 2024; Clark, 2024). In contrast, the moderating effect of general science was weakest, possibly because science courses have comprehensive characteristics, and when learning interdisciplinary topics like climate systems, which involve abstract models, students are prone to feelings of boredom, abstraction, and difficulty. This can diminish the effectiveness of their climate learning (Winarno et al., 2020). It should be noted that because the number of studies and effect sizes for chemistry, biology, and geography are far lower than those for earth science and general science, we need to treat the discipline ranking cautiously, viewing it as an exploratory finding. Nevertheless, despite differences in effect sizes, this meta-analysis shows that all science disciplines can significantly enhance students’ climate literacy. Therefore, continuing discipline-based science teaching related to climate change issues is worthwhile. Furthermore, some studies point out that relying solely on single-discipline approaches may not yield the best results (Salinas et al., 2022), so it is also necessary to explore multidisciplinary and interdisciplinary projects alongside integration within individual subjects to optimize climate change education.

Instructional strategy was not a significant moderator variable, indicating that the effectiveness of school science education in fostering students’ climate literacy is not contingent on whether digital technologies are used. On the one hand, this may be because climate change is a multi-layered issue that spans past, present, and future temporally and encompasses local, regional, and global scales spatially (USGCRP, 2024). Whether science classrooms connect students’ everyday experiences through traditional instructional approaches or simulate temperature trends using digital tools, students can experience strong contextual engagement (Igboanugo and Naiho, 2024; Mostacedo-Marasovic et al., 2023). On the other hand, this may reflect the fact that the effectiveness of technology depends heavily on how it is pedagogically integrated rather than on its mere presence (Wu, 2024). As long as the goals, content, and methods of climate literacy instruction are coherently aligned to actively engage learners, effective outcomes can be achieved (UNESCO, 2024). Existing research also indicates that both digital and traditional instructional approaches have their respective advantages in fostering students’ environmental-related literacies. Mulders and Träg (2023) found that extended reality (XR) could impart knowledge about biodiversity and influence students’ environmental attitudes. Labianca (2021) argued that integrating Geographic Information Systems (GIS) into geography teaching helps enhance local resource awareness. Boaventura et al. (2020) revealed that science teaching centered on inquiry-based experimental activities significantly enhanced students’ cognition and explanatory ability regarding the impacts of marine climate change. The study by Restović and Bulic (2024) showed that students’ stays and participation in environmental activities in national parks could significantly influence their positive attitudes towards nature conservation and environmental knowledge. These findings offer valuable insights into how we should navigate the relationship between digital empowerment and traditional teaching in the era of educational digitization. It should be noted that while the current dichotomous approach maximizes the robustness of meta-analysis results, it may obscure the heterogeneity of effects within similar strategies (e.g., among different digital tools). Therefore, this conclusion should be regarded as exploratory, and further research is needed to deepen our understanding of the roles played by different teaching strategies.

Educational level did not significantly moderate the impact of school science education on student climate literacy. This finding is consistent with Aeschbach et al. (2025), who found that educational level did not significantly moderate the effectiveness of climate change education across multiple dimensions including knowledge, attitudes, and behavior. We believe the lack of a significant moderating effect from educational level may stem from two aspects. On one hand, existing climate competency assessment scales may not have differentiated indicators designed for different educational levels, preventing the assessment results from reflecting true differences between educational levels (Horry et al., 2023). On the other hand, students across grade levels may generally lack the core cognitive abilities required to understand climate science, as revealed by Jin et al. (2013), who found that students from fourth to twelfth grade commonly held misconceptions about carbon emission mechanisms. For instance, they generally believed that gasoline “disappears” or “turns into energy” during combustion, overlooking the reaction with oxygen that produces carbon dioxide. It should be noted that due to the severe imbalance in sample sizes across educational attainment subgroups, the current findings should be regarded as exploratory and interpreted with caution.

Intervention duration was not a significant moderator variable, suggesting that the effectiveness of school science education in fostering students’ climate literacy is not proportional to the length of time. To date, research has not reached a consensus on this issue. A meta-analysis by Aeschbach et al.’s (2025) identified duration as a significant moderator of climate change education effects, and Sabarwal et al. (2024), based on an analysis of 96 countries worldwide, found that each additional year of education increases climate awareness by 8.6%. Çalik and Wiyarsi’s (2025) meta-analysis similarly showed that intervention duration significantly moderated the effectiveness of instruction on socioscientific issues. In contrast, Van De Wetering et al. (2022) reported that duration did not significantly moderate environmental education outcomes, and Doğan et al. (2023) found that the effectiveness of the flipped classroom approach in science education was not significantly moderated by duration. The present study corroborates the latter, non-significant findings. In this study, the lack of a significant moderating effect of intervention duration may be attributed to the fact that climate literacy is not a linearly cumulative learning outcome (Aeschbach et al., 2025), and its constituent sub-dimensions do not necessarily develop synchronously (Klement et al., 2025). Knowledge- and attitude-related outcomes concerning climate change may emerge within relatively short periods, while extending the duration of instruction may primarily serve to consolidate and deepen learning rather than proportionally amplify effect sizes. Thus, interventions lasting <1 month may already be sufficient to “initiate” climate literacy development (Pacini et al., 2025; Lombardi et al., 2013), and longer durations do not necessarily yield substantially higher average effects. For example, Pacini et al. (2025) employed only a four-hour instructional intervention and found that students’ environmental knowledge remained significantly higher than pre-intervention levels 4 weeks later. Lombardi et al. (2013) used a 90-min intervention and observed that students’ understanding of anthropogenic climate change remained stable 6 months after the intervention. Another possible explanation is that intervention quality matters more than duration. Short-term interventions (e.g., < 1 month) may benefit from focused and intensive climate change themes due to time constraints, while long-term interventions (e.g., more than 6 months) may suffer from unfocused themes and diluted content. It should also be noted that the number of studies and sample sizes varied substantially across duration subgroups (e.g., 16 studies in the “< 1 month” group versus only 7 studies in the “1–6 months” group), which may have limited the statistical power to detect true differences. Therefore, these findings should be interpreted with caution. Considering the potential limitations of the present study and the inconsistencies in existing research, the moderating role of intervention duration remains an important topic for further investigation.

## 5 Conclusions and recommendations

As a novel attempt to comprehensively analyze the impact of school science education on student climate literacy, this meta-analysis found that school science education exerts a significantly large and positive overall effect on student climate literacy. Sub-dimension analysis showed that school science education is most prominent in enhancing students’ climate change cognition, followed by attitude, while action did not reach significance. This study also found that discipline effectively moderates the impact of school science education on students’ climate literacy, while teaching strategy, educational level, and intervention duration are not key moderating factors.

Based on these findings, this study proposes four practical recommendations. First, systems thinking should be employed in top-level design to deeply integrate climate change topics into school science education. On one hand, integration should be organic and aligned with disciplinary characteristics. This meta-analysis confirms that all science disciplines contribute to developing student climate literacy, albeit with varying effectiveness. This suggests a need to both explore effective educational strategies grounded in specific disciplinary features. On the other hand, interdisciplinary collaborative projects should be developed. These should leverage the core strength of disciplines like chemistry in explaining climate change mechanisms, while synergistically utilizing the complementary advantages of geography, biology, and earth science in areas such as spatial distribution, ecosystems, and scientific observation. This approach transcends the limitations of single-discipline instruction, holistically enhancing students’ systemic understanding of and capacity to respond to climate issues. Second, a balanced and comprehensive cultivation of students’ climate change cognition, attitudes, and action should be emphasized. This meta-analysis reveals that cognitive outcomes yield the strongest effects, followed by attitudes, while actions remain insignificant. Given the volume of studies we included, the number of cognitive-related findings also far exceeds those concerning attitudes and actions. This suggests that current science education may exhibit an overemphasis on cognitive development when cultivating climate literacy. Therefore, while maintaining the cognitive strengths of science education, greater attention must be directed toward nurturing students’ attitudes, emotions, values, and actions regarding climate issues. Third, teaching strategies should be selected judiciously based on instructional objectives. Although teaching strategy was not a significant moderator variable, this meta-analysis found that both traditional teaching and science teaching using digital tools can effectively enhance students’ climate literacy. This insight suggests that when designing instruction integrating climate change topics, science teachers should not hold preconceived notions about any particular teaching strategy. Rather, they should recognize that any strategy serves as a means to achieve instructional goals and content. Whether to adopt traditional methods or integrate digital technology should be primarily determined by the goal of developing climate literacy. For example, cultivating ecological care is ideally achieved through nature experiences and field inquiry. Cultivating understanding of phenomena like surface temperature rise can be effectively addressed through computer climate modeling. Fourth, when designing and implementing climate change education programs, science teachers should shift their focus from increasing intervention duration to optimizing intervention design. This meta-analysis found that the effectiveness of science education in cultivating students’ climate literacy is not necessarily linked to intervention duration. This suggests that on one hand, the effects of short-term interventions should be valued; on the other hand, more effective long-term intervention strategies should be explored to avoid merely increasing time without improving effectiveness.

This study has several limitations. First, this meta-analysis only included studies published in English. However, given the global nature of climate change education, this may lead to an overestimation of the effect estimate. Furthermore, this study only included experimental and quasi-experimental research, potentially systematically excluding high-quality longitudinal or mixed-methods studies that more authentically capture behavioral changes. This design limitation may bias results towards cognitive gains. Future research should include studies in multiple languages and various research types to understand this phenomenon more comprehensively. Second, although this study’s three-dimensional division of climate literacy has a basis in the literature, it must be acknowledged that this classification has inherent limitations. For example, “cognition” may blur the distinction between knowledge and perception, and “attitude” may blur the distinction between values and emotions. As research on each sub-dimension increases, future meta-analyses need to classify more meticulously and cautiously. In this study, the action dimension had only three effect sizes, which not only made comparisons across the three dimensions difficult but also limited further moderator analysis by sub-dimension. Therefore, we recommend that future research increase attention to climate action. Third, due to the limitations of the included literature, this study only examined discipline, educational level, intervention duration, and teaching strategy as moderator variables, unable to test the potential influence of other relevant factors like teachers on students. Moreover, the moderator effect analysis for teaching strategy lacked more detailed analysis. This shortcoming stems from an insufficient number of studies on specific strategies, preventing us from establishing independent categories for effective analysis. Future research should explore the potential impact of other moderator variables on the effectiveness of school science education in cultivating students’ climate literacy more broadly and strive to focus more on teaching strategies for targeted exploration to better elucidate how various strategies differentially promote students’ climate literacy. Finally, given the significant heterogeneity among studies, the conclusions of this meta-analysis should be interpreted cautiously. It is recommended that future research adopt more unified methodological standards and conduct more experiments with high homogeneity to support more robust meta-analytic conclusions.

## Data Availability

The raw data supporting the conclusions of this article will be made available by the authors, without undue reservation.
